# In vitro and in vivo functional roles of three region 1 promoters in Escherichia coli K1 capsule gene cluster

**DOI:** 10.1099/mic.0.001783

**Published:** 2026-10-08

**Authors:** Esraa Aldawood, Ian S. Roberts

**Affiliations:** 1 Department of Clinical Laboratory Science, College of Applied Medical Science, King Saud University, P. O. Box 10219, Riyadh 11433, Kingdom of Saudi Arabia; 2 School of Biological Sciences, Faculty of Biology, Medicine and Health, Manchester Academic Health Science Centre, University of Manchester, Manchester, UK

**Keywords:** capsule expression, *Escherichia coli* K1, group 2 capsule, PR1 promoter regulation

## Abstract

Capsular polysaccharides are key virulence determinants in extraintestinal pathogenic *Escherichia coli*, protecting bacteria from host immune defences while modulating interactions with host tissues. In group 2 capsule gene clusters, transcription of region 1 genes required for capsule export is controlled by a complex regulatory region (PR1) containing three tandem promoters (PR1-1, PR1-2 and PR1-3).

In this study, targeted mutations were introduced into the −10 elements of each PR1 promoter in the uropathogenic strain UTI89. Transcriptional output from the PR1 region was assessed using a chromosomal PR1–*lacZ* fusion, and capsule expression was evaluated by K1 bacteriophage sensitivity assays, flow cytometry and immunofluorescence microscopy. Growth in Lysogeny Broth medium demonstrated that all three promoters contributed to capsule expression *in vitro,* with expression from both PR1-2 and PR1-3 being dependent on a functional upstream PR1-1.

In contrast, infection of PD07i bladder epithelial cells revealed a distinct regulatory pattern in which, following invasion, intracellular capsule expression was restored in the absence of PR1-1 and PR1-2, indicating that PR1-3 was sufficient for capsule induction within bladder cells. The presence of a predicted binding site for the oxygen-responsive regulator fumarate and nitrate reduction regulatory protein near PR1-3 suggests a possible mechanism for intracellular activation of PR1-3 under microaerobic host conditions in the absence of a functional PR1-1 promoter.

Together, these findings reveal a niche-dependent, multi-promoter regulatory architecture that enables adaptive control of capsule expression during infection.

## Data Summary

All data generated or analysed during this study are included in this published article and its supplementary information files. No large-scale datasets, genome sequences or custom code were generated. Strains and constructs used in this study are described in the Methods section.

## Introduction

Capsular polysaccharides are critical virulence factors in extraintestinal pathogenic *Escherichia coli*, contributing to severe infections, such as neonatal meningitis, septicaemia and urinary tract infections (UTIs) [1]. Among these, Group 2 capsules, including the K1 and K5 antigens, are particularly associated with invasive disease and enhanced bacterial survival within the host [2]. Capsule expression protects bacteria from innate immune defenses, such as complement-mediated killing and phagocytosis, while heterogeneous capsule expression within bacterial populations may enhance adaptability during infection [1–3].

Group 2 capsule gene clusters are organized into three distinct regions (Fig. 1) [4]. Regions 1 and 3 are highly conserved across all group 2 capsules and encode proteins required for polysaccharide export. In contrast, Region 2 is serotype-specific and contains genes responsible for the biosynthesis of the capsule polysaccharide and its associated precursors [5]. The promoter region PR1 is located 225 bp upstream of *kpsF* [6, 7] and contains three functional tandem promoters called PR1-1 (at +1), PR1-2 (at +132) and PR1-3 (at +181) that contribute to region 1 expression [8]. The PR3 promoter is located 741 bp upstream of the *kpsM* start codon [9]. An operon polarity suppressor sequence situated 28 bp upstream of *kpsM* (Fig. 1) enables RfaH-dependent transcriptional read-through from PR3, a process that is essential for transcription of the serotype-specific genes within region 2 [9–11]. Capsule expression is typically induced at 37 °C but repressed at 20 °C and this regulation is at the transcriptional level [7, 12]. Global regulators, such as H-NS, BipA, integration host factor (IHF) and SlyA modulate the PR1 and PR3 promoter activity in response to temperature [11–13] and growth phase [4, 14], while additional transcription factors, including fumarate and nitrate reduction (FNR), Fur, MprA and LrhA further fine-tune expression in response to oxygen and iron levels [15, 16].

**Fig. 1.**
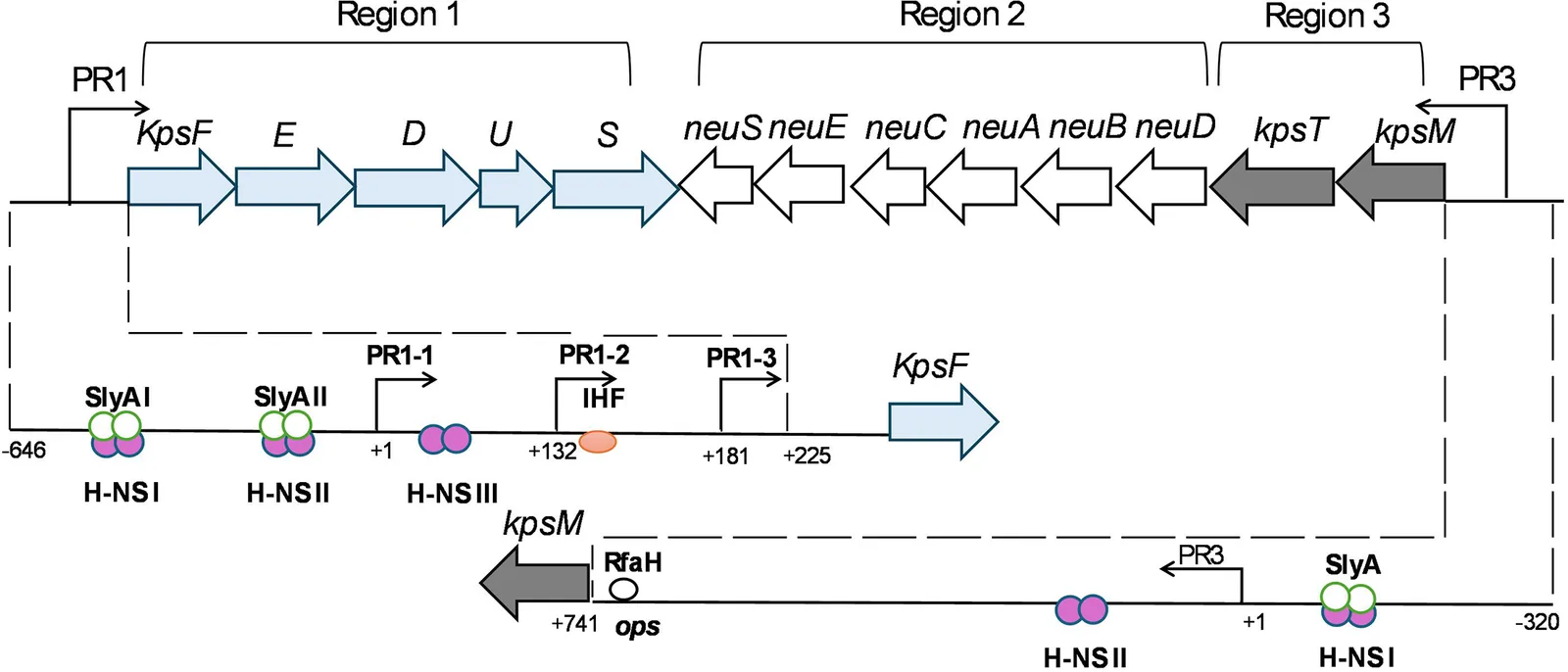
Genetic organization of the *E. coli* group 2 (**k1**) *kps* gene cluster. Group 2 *kps* clusters comprise three regions, with the conserved regions 1 and 3 flanking the serotype-specific region 2. Promoters driving transcription from regions 1 and 3 (PR1 and PR3, respectively) are indicated by bent arrows. PR1 directs transcription of region 1 genes, whereas PR3 initiates transcription of region 3 genes and enables read-through into region 2 via the RfaH antiterminator (white circle). The lower panel illustrates the regulatory architecture of the PR1 and PR3 promoters. The PR1 region contains three tandem promoters (PR1-1, PR1-2 and PR1-3). IHF (orange circle) binds to its consensus site at position +140, while H-NS (pink circles) binds to three consensus sites (H-NS I, II and III) spanning positions −224 to −134, –121 to −79 and +1 to +32, respectively. SlyA (white circles with green borders) and H-NS bind to the H-NS I and II sites upstream of PR1-1 in a non-mutually exclusive manner. At PR3, H-NS binds regions −135 to −21 and +141 to +219 relative to the transcription start site, with SlyA binding overlapping the upstream H-NS site. Nucleotide positions are shown relative to the respective transcription start sites. Adapted from [4].

*E. coli* expressing Group 2 capsules also display phenotypic heterogeneity such that individual bacterial cells within a population can exhibit variable capsule levels. Moreover, growth in urine leads to downregulation of capsule genes, suggesting that environmental conditions encountered during urinary tract colonization dynamically shape capsule production [3]. The observation that unencapsulated *E. coli* display higher adherence and invasiveness is likely to be critical in the early stages of infection. Whereas encapsulated cells are better protected from host intracellular defences at stages later in the infection process [3].

In the present study, we investigated the regulation of the three functional promoters within the PR1 region at the chromosomal level by introducing targeted mutations into the −10 elements of each promoter in a uropathogenic *E. coli* isolate (strain UTI89). The impact of these mutations on transcription originating from PR1 was assessed using a PR1–*lacZ* transcriptional fusion, and their effects on capsule expression were examined in the UTI89 background. Our results demonstrate that PR1-1 functions as the primary promoter during growth in Lysogeny Broth (LB) medium, activating transcription from PR1-2 and PR1-3, likely through transcription-coupled DNA supercoiling.

To investigate the role of the PR1 promoters in an intracellular environment, gentamicin protection assays were performed using UTI89 PR1 promoter mutants to infect PD07i bladder epithelial cells. In contrast to observations in LB medium, the coordinated promoter interactions observed under laboratory growth conditions were not maintained intracellularly. Instead, PR1-3 was both necessary and sufficient for capsule expression within bladder cells, functioning independently of PR1-1 and PR1-2, which were dispensable in this context.

Taken together, these findings indicate that the functional contribution of each PR1 promoter is niche-dependent, highlighting an additional layer of regulatory complexity within the PR1 region that enables adaptive capsule expression during different stages of infection.

## Methods

### Bacterial strains and culture conditions

The bacterial strains used in this study are listed in Table 1. Unless otherwise stated, strains were routinely cultured in LB medium at 37 °C. Where required, antibiotics were added at the following final concentrations: kanamycin (50 µg ml⁻¹), ampicillin (100 µg ml⁻¹) and chloramphenicol (25 µg ml⁻¹).

**Table 1.** Strains used in this study

Strain	Relevant information/genotype	Source/Reference
UTI89	Cystitis-derived isolate of serotype O18:K1:H7	[27]
UTI89P1*lacZ*	Chromosomal fusion of single copy *lacZ* gene inserted between *kpsF* and *kpsE* in UTI89Δ*lacZ*	[28]
UTI89Δ*lacZ*	*lacZ* deletion mutant of UTI89	[28]
FW-89	UTI89 with insertion of an inverted *kanR* cassette at −185 upstream of PR1-1	This study
F1-89	UTI89 with insertion of an inverted *kanR* cassette at −185 upstream of PR1-1 and a single-site substitution (T to C) at −13 bp of −10 hexamer of PR1-1.	This study
F2-89	UTI89 with insertion of an inverted *kanR* cassette at −185 upstream of PR1-1 and triple-site substitution (TATATT to TCTCTC) at −10 hexamer of PR1-2.	This study
F3-89	UTI89 with insertion of an inverted *kanR* cassette at −185 upstream of PR1-1 and substitution of ATAAATAAATTATA to CGTTCGTGTGCTGG at −10 hexamer of PR1-3.	This study
F1,2–89	UTI89 with insertion of an inverted *kanR* cassette at −185 upstream of PR1-1 and single-site substitution (T to C) at −13 bp of −10 hexamer of PR1-1 and triple-site substitution (TATATT to TCTCTC) at −10 hexamer of PR1-2.	This study
F2,3–89	UTI89 with insertion of an inverted *kanR* cassette at −185 upstream of PR1-1 and triple-site substitution (TATATT to TCTCTC) at −10 hexamer of PR1-2 and substitution of ATAAATAAATTATA to CGTTCGTGTGCTGG at −10 hexamer of PR1-3.	This study
FW-*lacZ*	UTI89P1*lacZ* with insertion of an inverted *kanR* cassette at −185 upstream of PR1-1	This study
F1-*lacZ*	UTI89P1*lacZ* with insertion of an inverted *kanR* cassette at −185 upstream of PR1-1 and single-site substitution (T to C) at −13 bp of −10 hexamer of PR1-1.	This study
F2-*lacZ*	UTI89P1*lacZ* with insertion of an inverted *kanR* cassette at −185 upstream of PR1-1 and triple-site substitution (TATATT to TCTCTC) at −10 hexamer of PR1-2.	This study
F1,2-*lacZ*	UTI89P1*lacZ* with insertion of an inverted *kanR* cassette at −185 upstream of PR1-1 and single-site substitution (T to C) at −13 bp of −10 hexamer of PR1-1 and triple-site substitution (TATATT to TCTCTC) at −10 hexamer of PR1-2.	This study
UTI89Δ*neu*	UTI89 Δ*neuDBACES*	[3]

### Construction of PR1 mutants

Chromosomal mutations were generated in the *E. coli* UTI89 background using the gene doctoring recombineering method described earlier [17]. Recombinants were selected on LB agar containing kanamycin and 5% (wt/v) sucrose and screened for loss of the donor plasmid and recombineering plasmid. Successful chromosomal integration of the mutations was confirmed by colony PCR using primers flanking the modified region and verified by DNA Sanger sequencing at the University of Manchester Genomic Technologies Core Facility.

The promoter mutations were designed based on previous functional characterization of PR1 regulatory region. For PR1-1 and PR1-2, previously validated mutations shown to disturb promoter activity were used [8]. In contrast, the functional −10 element of PR1-3 had not been conclusively defined. Previous mutagenesis of the predicted −10 sequence in a plasmid construct containing PR1-3 in isolation failed to abolish promoter activity, and previous 5′ RACE analysis (data not shown) indicated that isolating PR1-3 from its native regulatory context altered transcription start site selection. Therefore, a larger 14-nucleotide substitution was introduced within the native PR1 region to maximize disruption of PR1-3 activity while preserving the chromosomal promoter context.

### β-Galactosidase assays

Overnight cultures grown in LB with appropriate antibiotics were diluted 1 : 100 into fresh prewarmed LB and grown to mid-exponential phase (OD_₆₀₀_=1) and β-Galactosidase activity was measured as previously described [18].

### Cell line and bacterial infections

Human paediatric bladder epithelial cells (PD07i) were cultured as previously described [3]. Cells were maintained in serum-free EpiLife medium (Invitrogen) supplemented with 1% human keratinocyte growth supplement. PD07i cells were grown on sterile glass coverslips, then infected with bacteria in fresh EpiLife medium at a m.o.i. of 100. Plates were centrifuged at 500×g for 4 min to synchronize infection and incubated for 2 h at 37 °C with 5% (v/v) CO_₂_. After washing with PBS, fresh medium containing gentamicin (100 µg ml⁻¹) was added to eliminate extracellular bacteria, and cells were incubated until the indicated time points.

### K1 bacteriophage assay

Overnight cultures were grown at 37 °C to mid-exponential phase. Cells were harvested by centrifugation, resuspended in ice-cold 10 mM MgSO_₄_ and mixed with serially diluted K1 phage. After incubation at 30 °C for 30 min, mixtures were combined with soft LB agar (0.75% agar) and poured onto LB agar plates containing the appropriate antibiotics. Plates were incubated overnight at 37 °C.

### Immunoﬂuorescent microscopy

#### Bacterial cells

Bacterial cultures grown to mid-log phase (OD_₆₀₀_=1) in LB were washed, then fixed with 4% (v/v) paraformaldehyde, then washed three times with PBS and resuspended in PBS. Aliquots (10 µl) were spotted onto microscope slides and air-dried. Samples were blocked with 1% (wt/v) BSA in PBS and then incubated with an anti-K1 monoclonal antibody (IgG2a), raised against meningococcal group B and *E. coli* K1 capsular polysaccharides, which consist of identical α(2→8)-linked sialic acid homopolymers [19], (1 : 400) for 20 min at room temperature. After washing with PBS, samples were incubated with donkey anti-mouse IgG conjugated to Alexa Fluor 594 (1 : 500) and DAPI for 20 min. Slides were washed and mounted with Mowiol.

#### Infected PD07i cells

Infected bladder epithelial cells were washed with PBS and incubated with rabbit anti-*E. coli* IgG (1 : 200) for 30 min at 37 °C to label extracellular bacteria. Cells were then washed, fixed with 3% paraformaldehyde and permeabilized with 0.1% (v/v) Triton X-100 in PBS. Capsular polysaccharide was detected using anti-K1 antibody followed by donkey anti-mouse Alexa Fluor 594. Donkey anti-rabbit IgG Cy5 was used to detect anti-*E. coli* antibody, and nuclei were stained with DAPI. Samples were washed between steps and mounted with Mowiol.

Slides were examined using a Zeiss fluorescence microscope. Images were acquired with a Zeiss AxioImager D2 upright microscope using a 63×/1.4 EC Plan-Apochromat oil immersion objective and captured with a CoolSNAP HQ2 camera via Micro-Manager software (v1.4.23). Band-pass filter sets for DAPI and Texas Red were used to minimize channel bleed-through. Images were processed and analysed using ImageJ software.

### Flow cytometry analysis

Bacteria were grown in mid-log phase (OD_₆₀₀_=1) and ∼10^⁸^ cells were washed three times with PBS. Cells were blocked in 1% (wt/v) BSA–PBS for 1 h and incubated with anti-K1 antibody (1 : 400 dilution) in buffer A (1% (wt/v) BSA–PBS, 0.05% Tween 20) for 1 h at room temperature. After washing with buffer A, cells were incubated with donkey anti-mouse IgG conjugated to Alexa Fluor 594 (1 : 500 dilution) for 1 h at room temperature, followed by three additional washes. Fluorescence was measured by analysing 100,000 cells per sample using a BD Fortessa flow cytometer.

## Results

### Targeted −10 mutations reveal PR1-1 as the primary driver of PR1 transcription

Chromosomal fusions carrying targeted mutations in the −10 elements of the PR1 promoters were constructed in the UPEC strain UTIP1*lacZ* using the gene doctoring method. Four mutant strains containing the desired −10 promoter mutations were generated and designated FW, F1, F2 and F1,2, as illustrated in Fig. 2A.

**Fig. 2.**
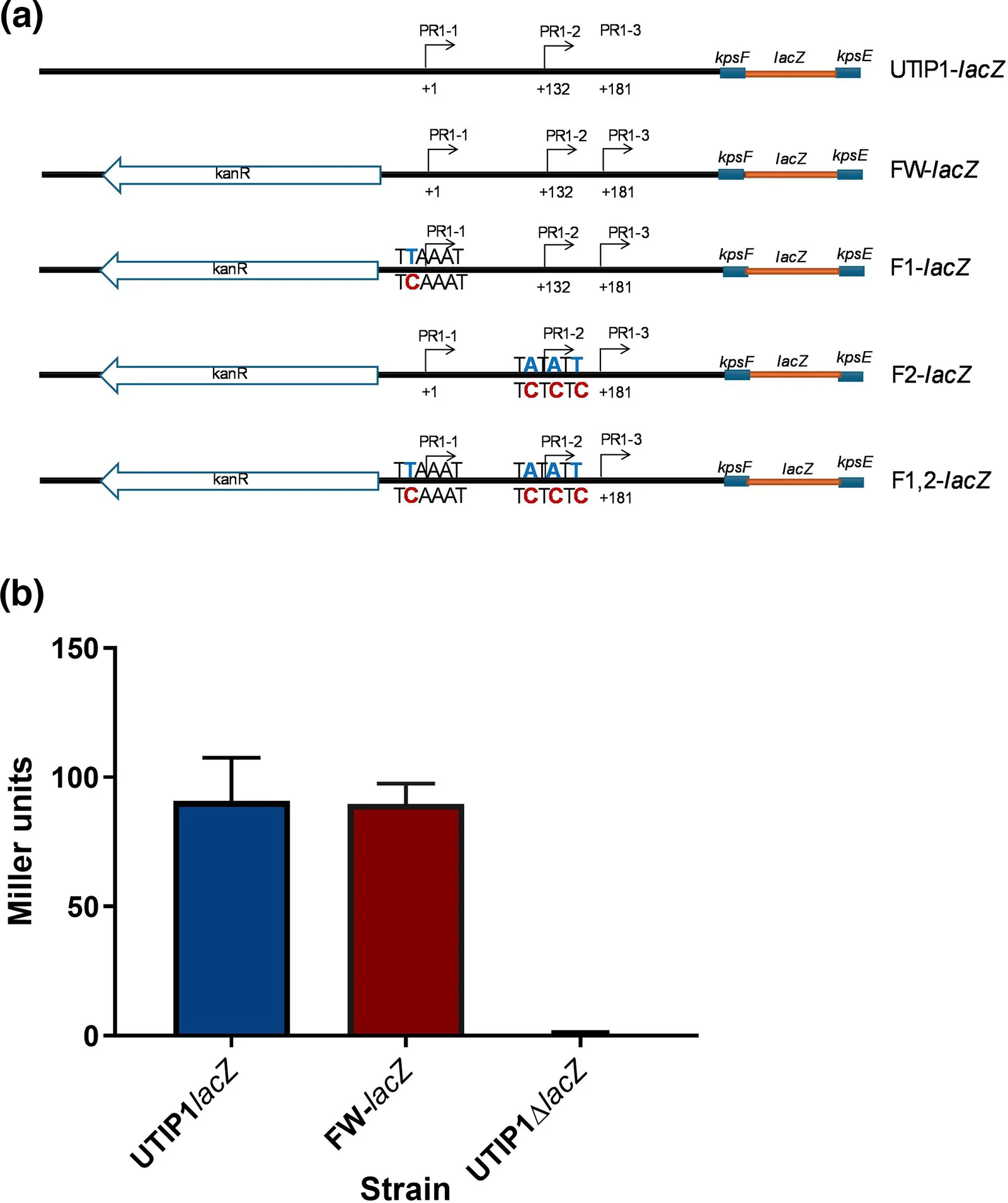
Construction of PR1 promoter mutants and assessment of kanamycin cassette insertion. (**a**) Schematic representation of the PR1–*lacZ* transcriptional fusion constructs used in this study. The UTIP1*lacZ* strain (previously described) carries a *lacZ* fusion between *kpsF* and *kpsE*. In the present study, an inverted kanamycin resistance cassette was inserted upstream of the PR1 region, and targeted mutations were introduced into the −10 elements of individual PR1 promoters. Bent arrows denote the transcription start sites. (**b**) Effect of kanamycin cassette insertion on transcription from PR1, assessed by measuring β-Galactosidase activity. Cultures of UTIP1*lacZ*, FW-*lacZ*, and UTIP1Δ*lacZ* strains were grown in LB at 37 °C to an OD_600_ of 0.5 prior to assay. Data represent the mean of three independent experiments performed in triplicate and are shown as mean±sem.

A PR1-3 −10 mutant (F3-*lacZ*) was also successfully constructed in UTIP1*lacZ* background and confirmed by DNA sequencing. However, unlike the corresponding mutation in the WT UTI89 background (see Section 6.2), the PR1-3 mutation did not reduce β-Galactosidase activity compared with the parental reporter strain.

Immunofluorescent microscopy likewise showed no obvious difference in capsule expression between FW-*lacZ* and F3-*lacZ* (Fig.S1). These findings suggest that the chromosomal *lacZ* insertion 3′ to the PR1 promoter region uniquely alters the regulation of the PR1-3 promoter, making this reporter background unsuitable for assessing PR1-3 activity. Therefore, the F3-*lacZ* strain was not included in the subsequent transcriptional analysis.

To determine whether insertion of a kanamycin resistance cassette upstream of PR1 in the opposite orientation influences transcription originating from the PR1 region, β-Galactosidase activity was measured in the FW-*lacZ* strain carrying the cassette and compared with UTIP1*lacZ*, which lacks the kanamycin insertion. The UTIP1Δ*lacZ* strain was included as a negative control. No significant difference in β-Galactosidase activity was observed between FW-*lacZ* and UTIP1*lacZ*, indicating that insertion of the kanamycin cassette upstream of the PR1 promoters does not affect transcriptional output from region 1 (Fig. 2B).

All PR1 promoter mutants displayed growth rates comparable to the FW-*lacZ* strain (Fig. 3A), indicating that the introduced mutations did not affect bacterial growth. To measure transcription coming from each promoter in the PR1 region, β-Galactosidase assays were performed at multiple stages of the growth curve for all strains (Fig. 3B). The most pronounced effect was a substantial reduction in transcriptional activity upon mutation of PR1-1, as observed in the F1-*lacZ* and F1,2-*lacZ* strains. Comparison of F2-*lacZ* with FW-*lacZ* demonstrated that PR1-2 contributes ∼50% of total PR1-dependent transcription throughout all growth phases, but this activity is dependent on a functional upstream PR1-1 (Fig. 3).

**Fig. 3.**
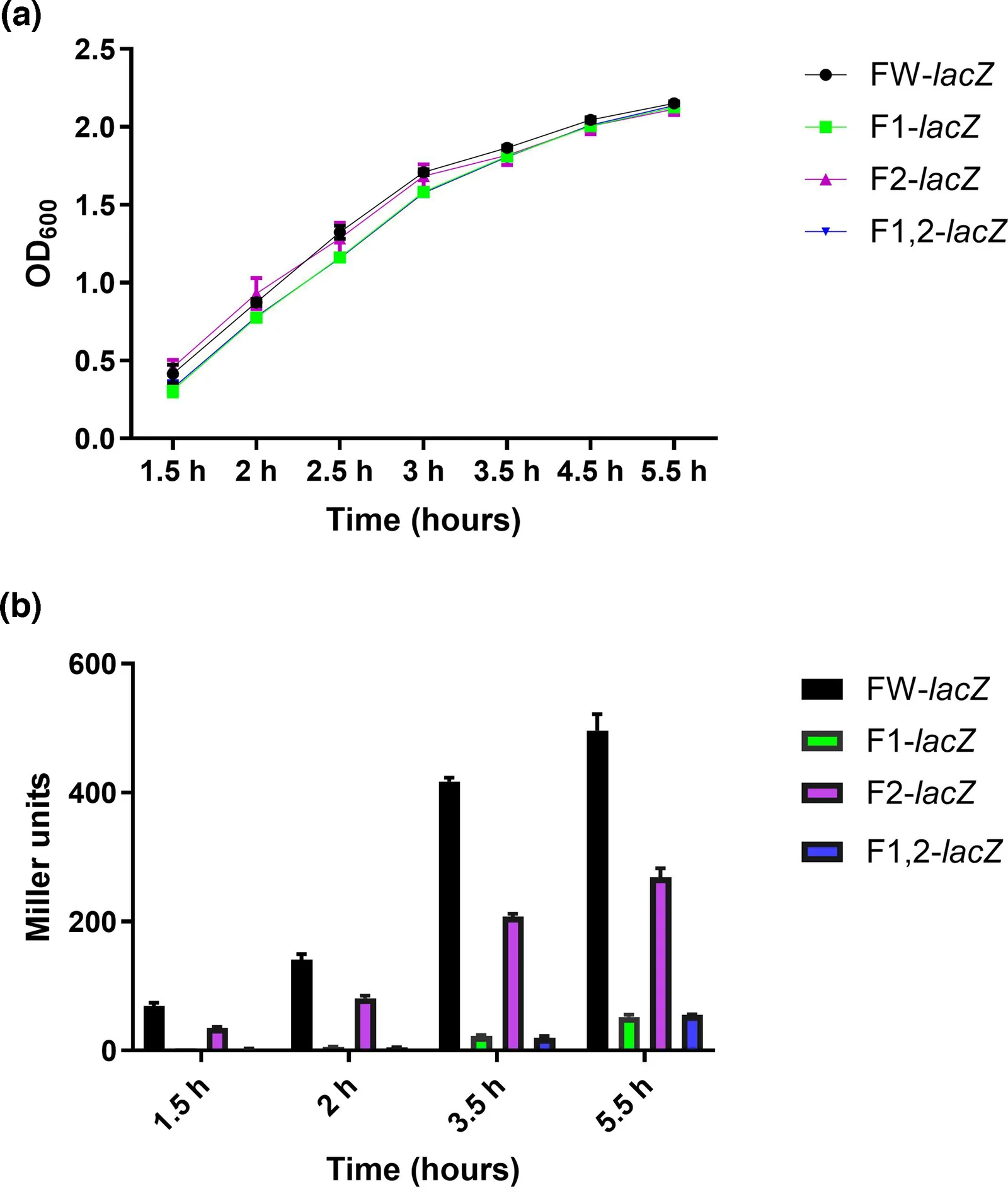
PR1 transcriptional activity of PR1 promoter mutants. (**a**) Growth curves of all strains cultured in LB at 37 °C. (**b**) β-Galactosidase activity of each PR1–*lacZ* construct measured at defined stages of the growth curve. Data represent the mean of three independent experiments performed in triplicate and are shown as mean±sem.

### PR1-1 controls capsule expression, whereas PR1-2 and PR1-3 promote uniform capsule expression across the bacterial population

Mutations in the −10 elements of PR1-1 and PR1-2, either individually or in combination, affected transcriptional output from the PR1 region. To determine whether these transcriptional effects were reflected at the level of capsule production, the same targeted −10 promoter mutations – together with an additional −10 mutation in PR1-3 – were reconstructed in the WT UTI89 background, generating strains FW-89, F1-89, F2-89, F1,2–89, F3-89 and F2,3–89 (Fig. 4A). K1 capsule expression in these strains was then assessed using multiple assays.

**Fig. 4.**
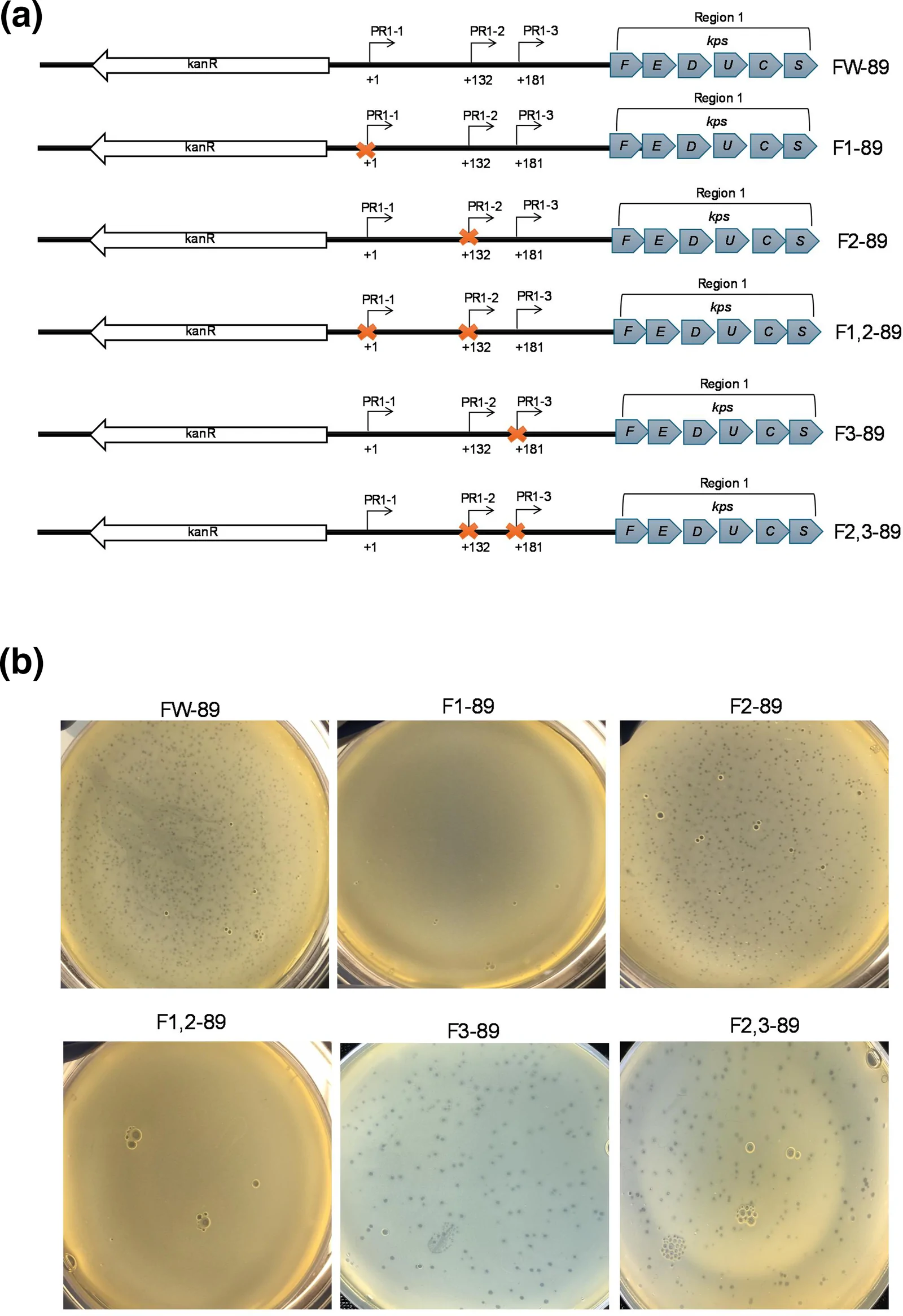
Effect of PR1 promoter mutations on susceptibility to K1-specific bacteriophage. (**a**) Schematic representation of PR1 promoter mutant constructs generated in the UTI89 background. An inverted kanamycin resistance cassette was inserted upstream of the PR1 region. Red crosses indicate targeted mutations in the −10 elements of individual PR1 promoters, and bent arrows denote transcription start sites. (**b**) Phage sensitivity assay of PR1 promoter mutant strains. Cultures were grown in LB at 37 °C to mid-exponential phase (OD_₆₀₀_=1) prior to exposure to K1-specific bacteriophage. The experiment was performed independently three times with similar results.

Capsule production was first evaluated using a plaque assay with a K1-specific bacteriophage. Strains F2-89 (PR1-2 −10 mutation), F3-89 (PR1-3 −10 mutation) and F2,3–89 (PR1-2/PR1-3 −10 mutations) remained susceptible to K1-specific bacteriophage and produced plaques comparable in appearance to those observed for the WT strain (FW-89) (Fig. 4B). In contrast, strains carrying a −10 mutation in PR1-1, either alone (F1-89) or in combination with a PR1-2 mutation (F1,2–89), were resistant to K1-specific phage infection, indicating a loss of functional capsule expression (Fig. 4B).

To validate the plaque assay findings, capsule expression was further examined by flow cytometry using an anti-K1–specific monoclonal antibody. All strains were grown in LB at 37 °C to mid-exponential phase (OD_₆₀₀_=1) prior to analysis. A capsule-deficient UTI89 mutant lacking region 2 synthesis genes (UTI89Δ*neu*) [16] served as the negative control.

In the WT strain (FW-89), the majority of cells (84%) were encapsulated, with only 16% lacking detectable capsule. In contrast, the F1-89 and F1,2–89 mutants displayed a pronounced leftward shift comparable to the UTI89Δ*neu* control, indicating that mutation of PR1-1 markedly reduces surface capsule expression (Fig. 5). Mutation of either PR1-2 (F2-89) or PR1-3 (F3-89) resulted in an approximately threefold reduction in the proportion of encapsulated cells relative to the WT (Fig. 5). Similarly, mutations of PR1-2 and PR1-3 in combination (F2,3–89) led to a threefold decrease in encapsulation compared to FW-89.

**Fig. 5.**
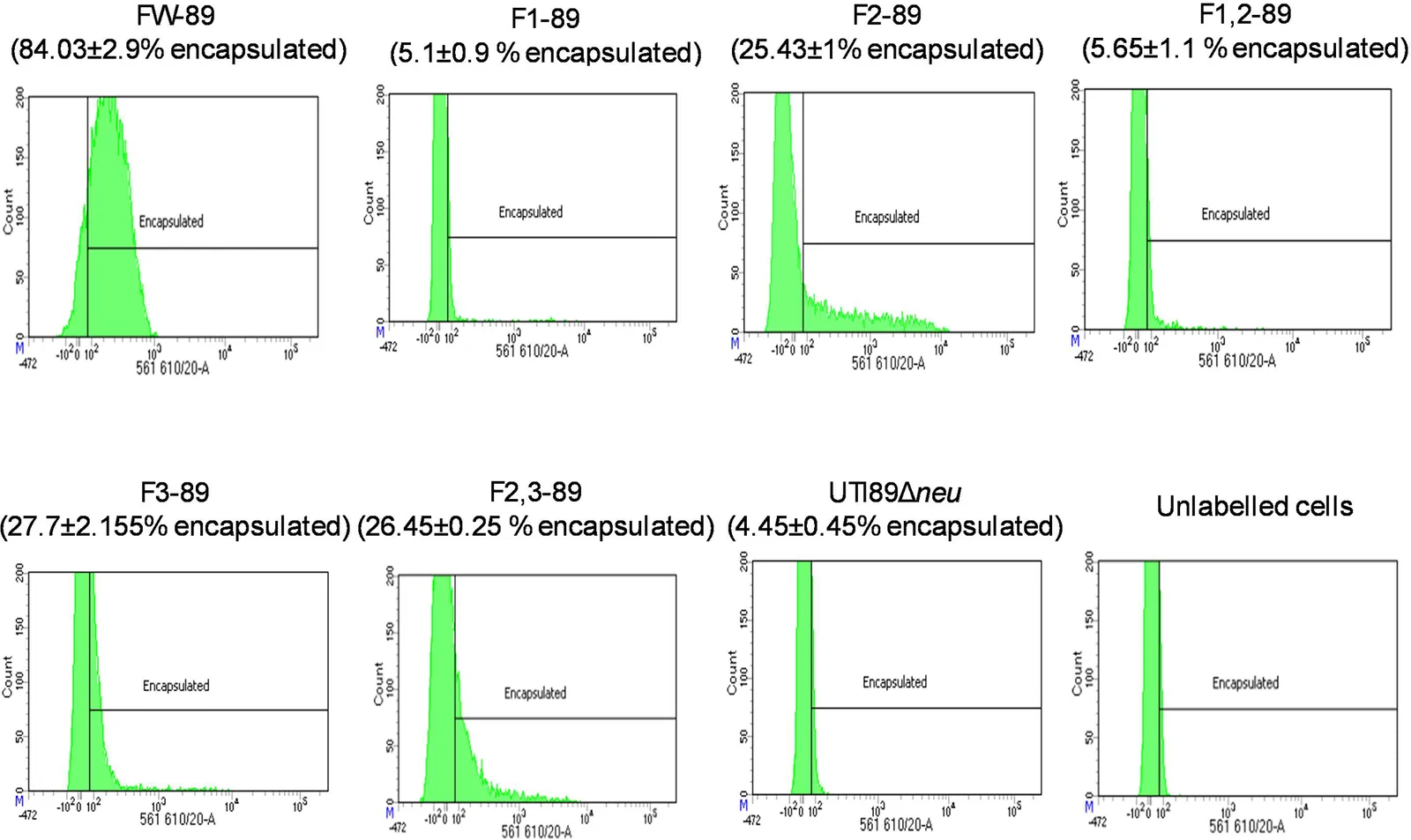
Flow cytometry analysis of capsule expression in UTI89 PR1 promoter mutants. Cultures of all strains were grown in LB at 37 °C to mid-exponential phase (OD_₆₀₀_=1) and stained with anti-K1 primary antibody, followed by AF594-conjugated secondary antibody and DAPI. Samples were analysed by flow cytometry, with the x-axis representing fluorescence intensity and the y-axis representing cell count. Encapsulated cells were gated by subtracting the signal from unstained controls. Percentages of encapsulated cells are indicated above each histogram. Data represent the mean of three independent experiments and are shown as mean±sem.

A small, highly fluorescent subpopulation was consistently observed within the F2-89 mutant but was absent from the WT strain. To investigate this observation, the unencapsulated, encapsulated and highly fluorescent populations were isolated by fluorescence-activated cell sorting and regrown prior to repeat flow cytometric analysis. Whereas the unencapsulated and encapsulated populations reverted to the original F2-89 phenotype, the highly fluorescent population consistently exhibited a WT-like capsule expression profile (Fig. S2). Sequencing confirmed that the engineered PR1-2 mutation remained intact and that the PR1 and PR3 promoter regions were unchanged, suggesting that the highly fluorescent population arose from a suppressor mutation elsewhere in the chromosome.

To assess capsule distribution at the single-cell level, FW-89 and mutant strains were grown to mid-exponential phase and analysed by immunofluorescence microscopy using an anti-K1 antibody. WT cells were predominantly fully encapsulated, with a small unencapsulated subpopulation, consistent with flow cytometry data (Fig. 6). In contrast, −10 mutations in PR1-1 (F1-89) and in both PR1-1/1-2 (F1,2–89) abolished uniform surface capsule expression, although occasional localized accumulations of polysaccharide were observed at the bacterial surface (Fig. 6), consistent with impaired capsule export rather than complete loss of polysaccharide synthesis. Mutations in PR1-2 and/or PR1-3 reduced the proportion of encapsulated cells, with ∼25% of bacteria remaining fully encapsulated, in agreement with flow cytometry results. As expected, the capsule-null mutant UTI89Δ*neu* showed no antibody binding.

**Fig. 6.**
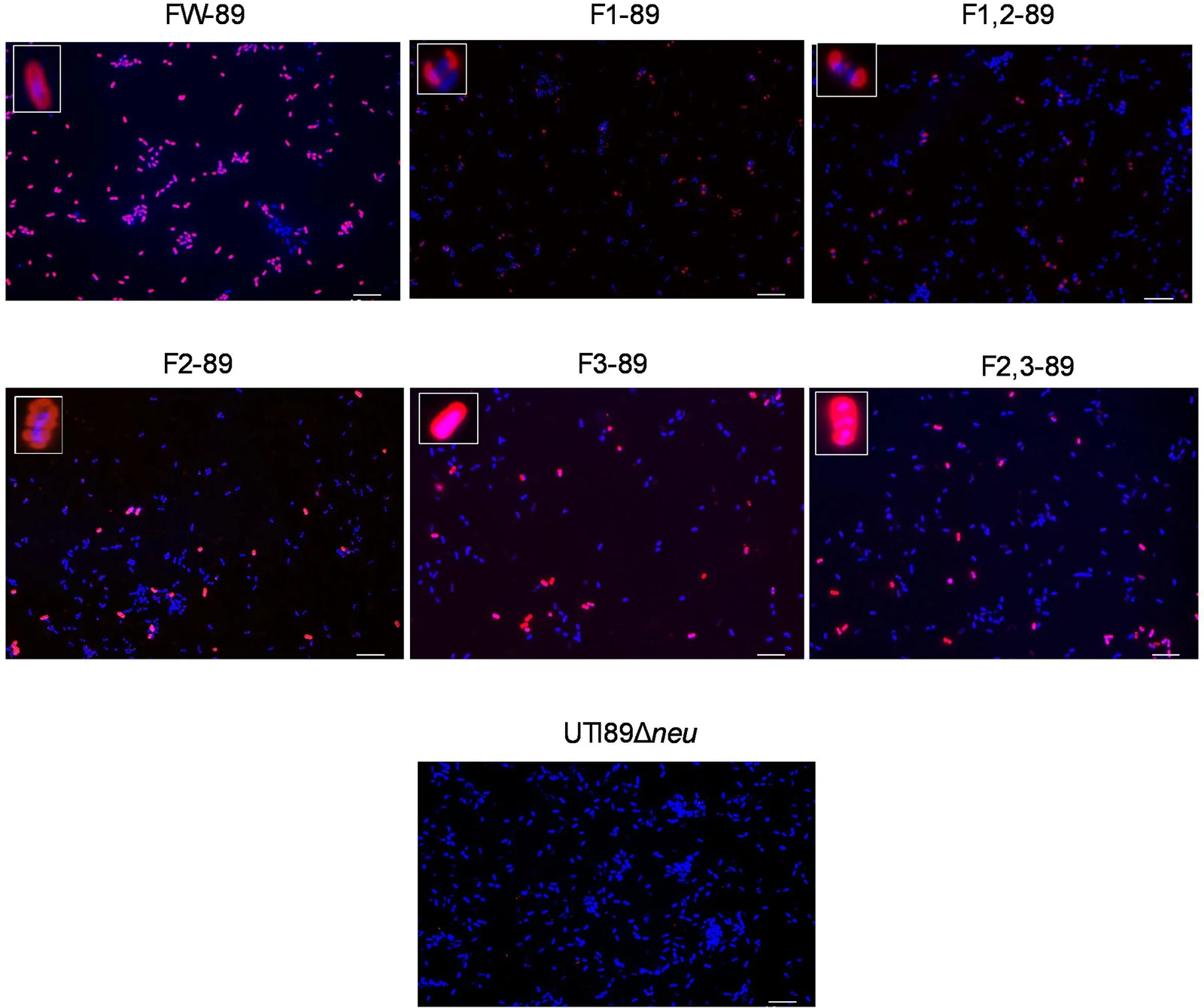
Immunofluorescence analysis of capsule expression in UTI89 PR1 promoter mutants. Strains were grown in LB at 37 °C to mid-exponential phase (OD_₆₀₀_=1), fixed with 4% paraformaldehyde and stained with anti-K1 primary antibody and Alexa Fluor 594-conjugated secondary antibody, with DAPI for nucleic acid visualization (see Methods). Encapsulated bacteria appear pink, while unencapsulated bacteria appear blue. Scale bar=10 µm. Insets show enlarged views of encapsulated cells.

Taken together, these results indicate that mutation of PR1-1 abolishes capsule expression in all cells and is absolutely required for encapsulation and K1 expression *in vitro*. In contrast, mutation of PR1-2 or PR1-3 markedly reduces the proportion of encapsulated bacteria, with approximately one quarter of cells remaining fully encapsulated. This indicates that in the presence of a functional PR1-1, these two additional promoters contribute to capsule expression *in vitro* but cannot function in the absence of PR1-1 to drive transcription of region 1 and capsule expression.

### PR1-3 is responsible for encapsulation inside PD07i bladder cells while PR1-1 seems to be redundant

It is known that unencapsulated UTI89 bacteria preferentially initiate bladder cell colonization, followed by K1 capsule gene upregulation after intracellular entry [3]. To determine whether the three PR1 promoters contribute to the induction of K1 capsule expression following entry into bladder cells, a gentamicin protection assay was first performed using the WT FW-89 strain to establish the timing of capsule upregulation following invasion prior to testing PR1 mutants. PD07i bladder cells were infected for 2 h, extracellular bacteria were eliminated with gentamicin and capsule expression was monitored at multiple time points by immunofluorescence microscopy. At 2 h post-gentamicin treatment, intracellular bacteria were predominantly unencapsulated, whereas extracellular bacteria were fully encapsulated, confirming that unencapsulated cells act as initial invaders (Fig. S3). At later time points (4–24 h), capsule expression appeared in a subset of intracellular bacteria. These findings confirm that capsule upregulation occurs after intracellular entry in a subpopulation of bacteria. Our hypothesis was that the multiple functional promoters within the PR1 region may contribute to this intracellular capsule induction.

To test this hypothesis, PD07i bladder cells were infected with PR1 promoter mutants alongside the WT (FW-89) and the capsule-null mutant (UTI89Δ*neu*). Because the timing of intracellular capsule induction could differ among mutants, capsule expression was assessed 24 h post-gentamicin treatment (Fig. 7). Strikingly, intracellular bacteria in the PR1-1 mutant (F1-89) and the PR1-1/PR1-2 double mutant (F1,2–89) retained capsule expression, indicating that PR1-3 can function independently within bladder cells (Figs. 7 and 8). Quantitative enumeration of intracellular bacteria at 24 h post-gentamicin treatment from a representative infection experiment is provided in Table S1. In contrast, mutation of PR1-3 (F3-89) or combined mutation of PR1-2 and PR1-3 (F2,3–89) markedly impaired intracellular capsule expression, whereas mutation of PR1-2 alone (F2-89) had no effect on capsule expression, suggesting that PR1-2 is dispensable intracellularly.

**Fig. 7.**
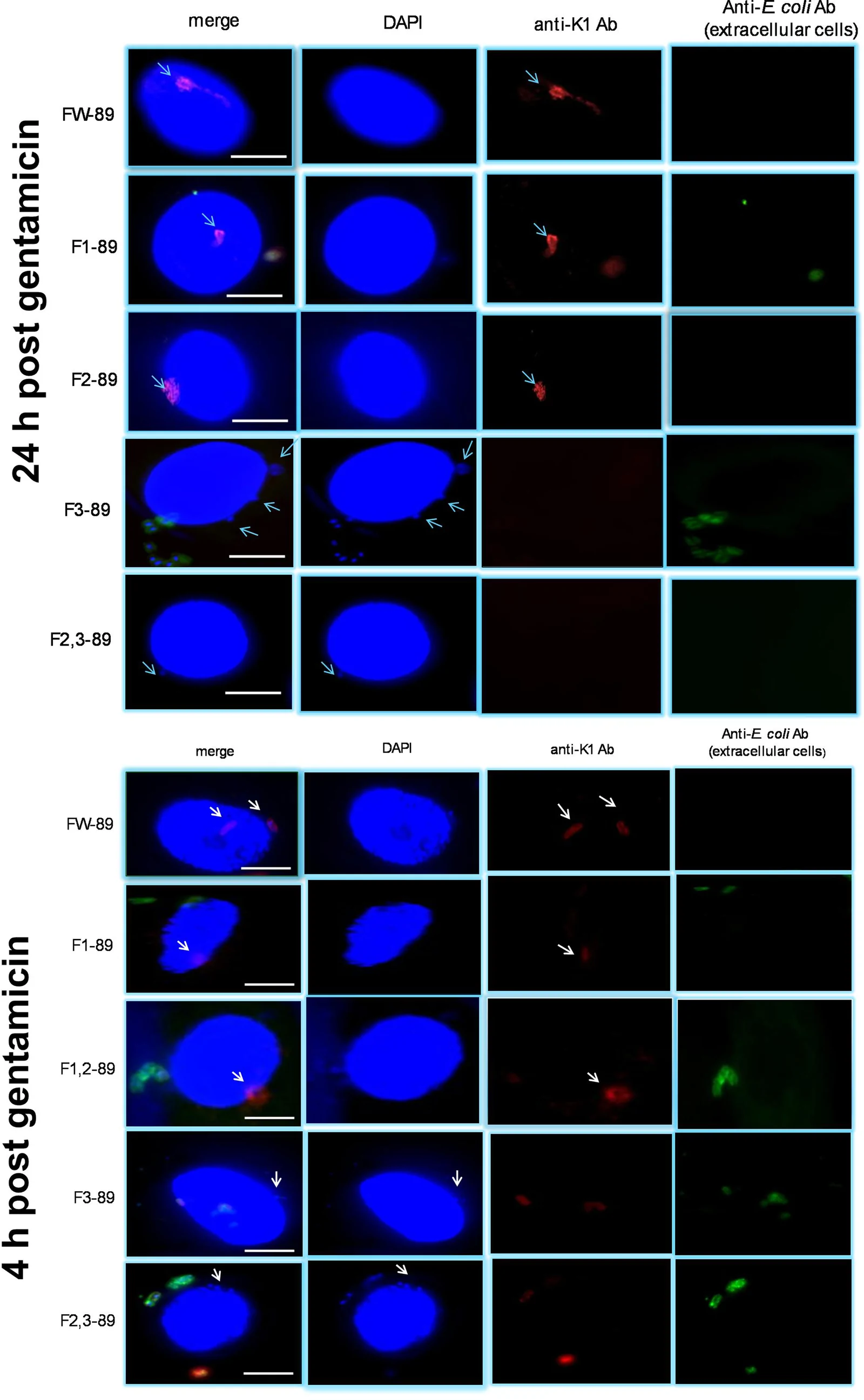
Effect of PR1 mutations on intracellular capsule expression in PD07i bladder cells. PD07i cells were infected with the indicated strains for 2 h, followed by gentamicin treatment to eliminate extracellular bacteria. Cells were incubated for either 4 h or 24 h post-treatment before staining. Extracellular bacteria were labelled with anti-*E. coli* antibody prior to fixation. Following permeabilization, intracellular bacteria were stained with anti-K1 antibody, DAPI and appropriate secondary antibodies (see Methods). Fluorescence microscopy images show individual channels and the merged composite. Scale bar=10 µm. Green arrows indicate intracellular unencapsulated bacteria. The experiment was performed at least three times, screening the entire slide for intracellular events.

**Fig. 8.**
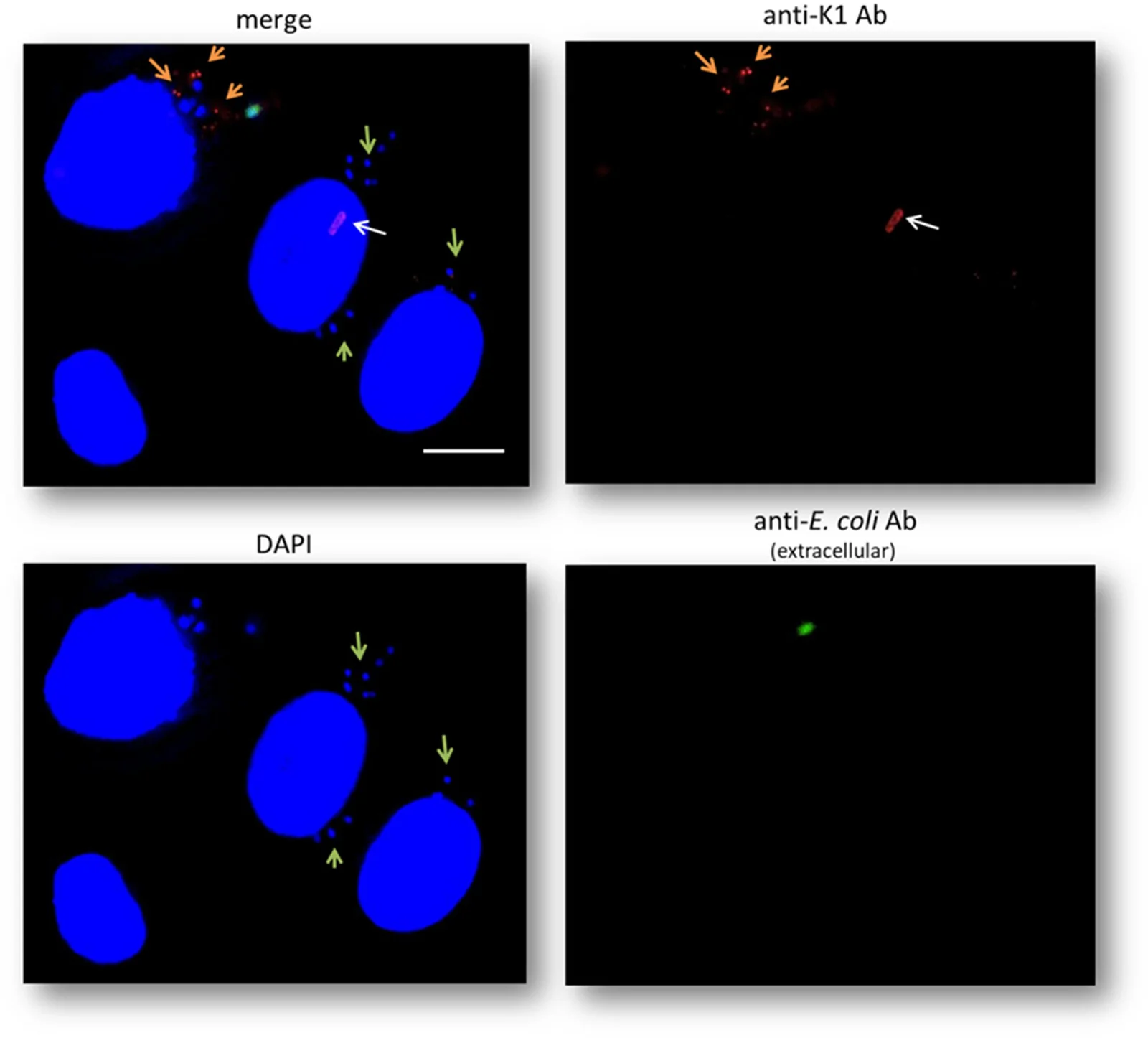
F1-89 strain expresses capsule inside PD07i cells 24 h post-gentamicin. PD07i cells were infected with F1-89 for 2 h, treated with gentamicin to remove extracellular bacteria and incubated for 24 h before staining. Extracellular bacteria were labelled prior to fixation, followed by permeabilization and staining with anti-K1, DAPI and secondary antibodies. Fluorescence microscopy images show representative fields with encapsulated (white arrows) with the coexistence of unencapsulated intracellular bacteria (green arrows) and partially encapsulated bacteria (orange). Scale bar=10 µm.

To assess the timing of PR1-3–driven capsule expression in bladder cells and whether PR1-1 or PR1-2 upregulate capsule genes early, a gentamicin protection assay was performed as described above, with cells labelled 4 h post-gentamicin. As shown in Fig. 7, results were comparable to those at 24 h post-gentamicin. Quantitative enumeration at 4 h post-gentamicin treatment is provided in Table S2. As a control, bacteria pre-diluted in Epilife media were incubated under the same conditions without bladder cells or gentamicin. Fig. S4 shows that WT bacteria were fully encapsulated, whereas F1-89 and F1,2–89 displayed only polysaccharide bulbs, indicating that capsule formation in bladder cells depends on bacterial internalization rather than media incubation alone.

To verify that the intracellular encapsulation observed at 4 h post-gentamicin in F1-89, F1,2–89 and F2-89 was not due to pre-encapsulated bacteria invading bladder cells, PD07i cells were examined 2 h post-gentamicin. The intracellular bacteria, including WT, were unencapsulated at this time, indicating that capsule expression occurs only after bacterial internalization (Fig. S5).

Overall, the bladder infection data indicate that PR1-3 acts as an independent promoter, enabling full bacterial encapsulation inside bladder cells, unlike its *in vitro* phenotype. PR1-1 and PR1-2 appear dispensable in this context.

## Discussion

Capsule production in UPEC strains protects bacteria from phagocytosis [20] but may reduce adhesion to bladder epithelial cells [3]. The three promoters in the PR1 region, potentially regulated by different factors, allow rapid adaptation by modulating capsule gene expression according to environmental conditions [4]. Notably, around 25% of *E. coli* transcriptional units have multiple promoters, which can act as backups and provide a fitness advantage in case of deleterious mutations [21, 22]

The heterogeneous capsule expression observed in the F2-89 population further highlights the dynamic nature of capsule regulation. Although the majority of F2-89 cells exhibited reduced capsule expression following disruption of PR1-2 activity, a small highly fluorescent subpopulation with a WT-like capsule phenotype was consistently observed. Isolation and characterization of this population demonstrated that the engineered PR1-2 mutation remained unchanged, suggesting that this phenotype did not result from restoration of PR1-2 promoter activity. Instead, the emergence of this population is consistent with a secondary suppressor mutation elsewhere in the chromosome that compensates for reduced PR1-2-dependent capsule expression. This finding suggests that additional regulatory pathways may contribute to maintaining capsule production under conditions where capsule expression provides a selective advantage.

*In vitro* experiments in LB demonstrated that PR1-1 functions as the major promoter controlling capsule expression. Mutations in PR1-1 (F1-89) or in both PR1-1 and PR1-2 (F1,2–89) markedly reduced surface capsule expression, confirming that PR1-1 is required for efficient activation of downstream promoters.

In contrast, mutation of either PR1-2 or PR1-3 reduced the proportion of encapsulated cells without completely abolishing capsule expression, indicating that these promoters contribute to maintaining capsule expression across the bacterial population rather than initiating it independently. This interpretation suggests that PR1-2 and PR1-3 reinforce or sustain transcription once initiated by PR1-1, thereby increasing the proportion of encapsulated cells under laboratory growth conditions. Interestingly, simultaneous mutation of PR1-2 and PR1-3 did not further reduce the proportion of encapsulated cells compared with either single mutant. The lack of an additive effect suggests that PR1-2 and PR1-3 may function within the same regulatory pathway downstream of PR1-1, such that disruption of either promoter is sufficient to reduce capsule expression in a substantial proportion of the population.

These findings are partially consistent with the model proposed by Jia *et al*. (2017), who reported that transcription from PR1-2 depends on PR1-1 through transcription-coupled DNA supercoiling, while PR1-3 can act independently [8]. However, in the present study, PR1-3 also appeared to depend on PR1-1 for efficient capsule expression. A likely explanation for this discrepancy is methodological: the earlier study examined promoter activity using multicopy plasmids in which PR1-3 was cloned independently of the surrounding promoter region. In contrast, the chromosomal context examined here suggests that isolating PR1-3 may create an artificial promoter configuration that does not exist naturally. This interpretation is supported by transcription start site mapping using RACE analysis (data not shown), which indicates that the native promoter architecture differs from that observed in plasmid constructs [14].

An additional discrepancy was observed between the chromosomal PR1–*lacZ* reporter strain and the WT UTI89 background for the PR1-3 mutation. Whereas mutation of PR1-3 reduced capsule expression in the WT background, the corresponding mutation in the PR1–*lacZ* reporter strain did not alter either β-Galactosidase activity or capsule expression. These findings indicate that the chromosomal PR1–*lacZ* reporter does not fully recapitulate native PR1-3 regulation, limiting its suitability for evaluating PR1-3 activity. Consequently, conclusions regarding PR1-3 function were based primarily on the WT UTI89 background.

The regulatory behaviour of the PR1 promoters appears to differ in bladder epithelial cells. Infection experiments demonstrated that capsule expression can be restored intracellularly even when PR1-1 and PR1-2 are mutated, indicating that PR1-3 can drive capsule expression inside host cells. This suggests that additional host-associated regulatory signals may activate capsule transcription during intracellular infection. One possible regulator is FNR (regulatory protein), a transcription factor that responds to low-oxygen environments. Previous work showed that FNR binds within the untranslated region of the PR1 regulatory region [15].

Following a thorough analysis (Fig. S6), the predicted FNR binding site was found to overlap three nucleotides of the −10 element of PR1-2 and lies upstream of PR1-3. Because only the second nucleotide of the PR1-2 −10 element was altered in the PR1-2 mutant, disruption of FNR binding is unlikely. FNR typically binds as a dimer centred around −41 relative to the transcription start site to activate transcription through direct interaction with RNA polymerase [23, 24]. Mapping of the predicted binding site indicates that it lies ∼+115 relative to the PR1-1 transcription start site and only two bases upstream of PR1-2, positions unlikely to support activation of these promoters. In contrast, the site is located ∼−52 relative to the PR1-3 transcription start site, a position compatible with FNR-dependent promoter activation. Therefore, PR1-3 represents the plausible candidate promoter for FNR-mediated regulation. However, FNR is not the only possible mechanism by which PR1-3 may be activated during intracellular infection. The specific intracellular compartment occupied by UTI89 within PD07i bladder epithelial cells was not determined in this study. Although our microscopy approach distinguishes intracellular from extracellular bacteria, it does not resolve the precise intracellular niche. Different intracellular environments may expose bacteria to distinct regulatory signals, including oxygen and nutrient availability, which could influence capsule expression. Iron availability represents another potential environmental signal affecting capsule regulation. However, previous work by Ma *et al*. examined Fur binding to the *kps* regulatory region and did not identify direct Fur binding within the PR1 regulatory region. Therefore, current experimental evidence does not support direct Fur regulation of PR1-3. Nevertheless, indirect effects of iron availability on capsule expression during intracellular growth cannot be excluded.

Given that intracellular environments, such as bladder epithelial cells can be relatively microaerobic [25, 26], it is plausible that FNR enhances capsule transcription from PR1-3 during infection. Further investigation of FNR-dependent regulation of the PR1 region would, therefore, help clarify the mechanisms controlling capsule expression during host colonization. Additionally, future studies examining capsule heterogeneity across different growth phases will help determine whether the contribution of individual PR1 promoters changes during bacterial growth i*n vitro*.

In summary, this study demonstrates that PR1-1 serves as the primary driver of capsule expression *in vitro*, while PR1-3 independently mediates intracellular capsule induction. These findings underscore the complex regulatory architecture of the PR1 region, enabling UPEC to adapt rapidly to environmental cues and host defences. Future studies should investigate the role of regulators, such as FNR in modulating PR1-3 activity and explore anti-capsule strategies as potential therapeutic interventions.

## Supplementary material

10.1099/mic.0.001783 Supplementary Material 1.

## Abbreviations

FNR: fumarate and nitrate reduction
IHF: integration host factor
LB: Lysogeny Broth
UTI: urinary tract infection
