# Supplementary material for: In vitro and in vivo functional roles of three region 1 promoters in Escherichia coli K1 capsule gene cluster
Source: Microbiology (Reading). 2026 Oct 8;172(10):001783. doi: 10.1099/mic.0.001783 (PMC13656800; doi:10.1099/mic.0.001783)
Supplement: Supplementary file 1 [file mic-172-01783-s001.pdf]

In Vitro and *In Vivo* Functional Roles of Three Region 1 Promoters in *Escherichia coli* K1 capsule gene cluster.

<u>The Supplementary Material</u>

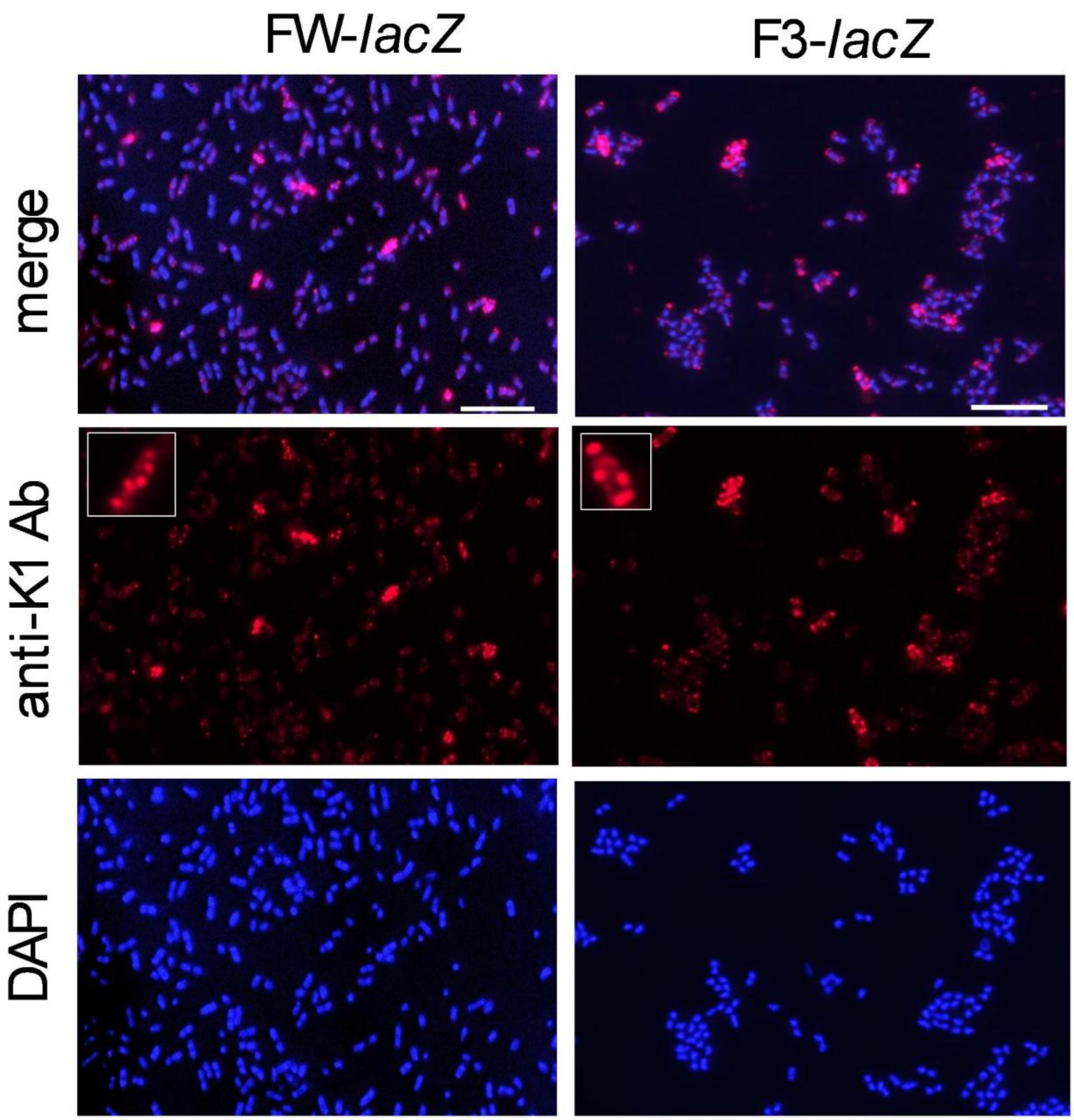

Figure S1 Mutation of PR1-3 does not alter capsule expression in the PR1–lacZ reporter background. FW-lacZ and F3-lacZ strains were grown in LB at 37°C to mid-exponential phase (OD_{600} = 1), fixed with paraformaldehyde, and stained with anti-K1 primary antibody followed by Alexa Fluor 594-conjugated secondary antibody and DAPI. Capsule-positive bacteria appear pink, whereas

unencapsulated bacteria appear blue. Individual fluorescence channels and merged images are shown for each strain. Insets show enlarged views of representative encapsulated cells. Scale bar = 10 µm.

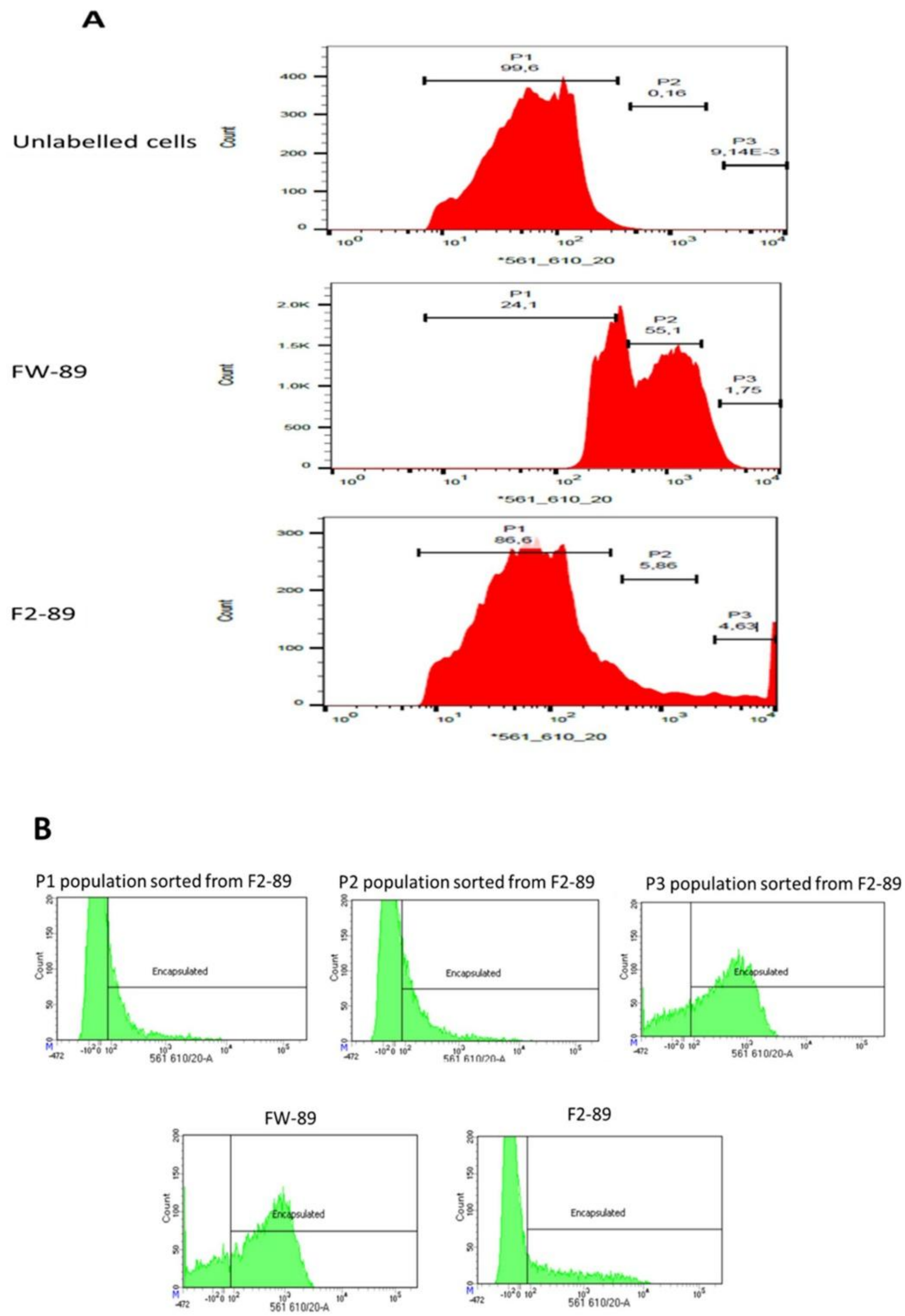

Figure S2 The sorted minor population in the F2-89 strain exhibits a wild-type-like capsule expression phenotype. (A) Representative flow cytometry histograms of pre-sorted FW-89 and F2-89 populations grown to mid-exponential phase (OD_{600} = 1) and stained with anti-K1 antibody followed by

Alexa Fluor 594-conjugated secondary antibody before sorting. P1 represents the unencapsulated population, P2 the predominant encapsulated population, and P3 the minor highly fluorescent population detected in F2-89. The percentage of cells within each gate is indicated. Sorted populations were reanalysed to confirm sorting accuracy. (B) Sorted F2-89 populations were regrown, stained, and analysed by flow cytometry alongside FW-89 and F2-89 strains. Data represent three independent experiments.

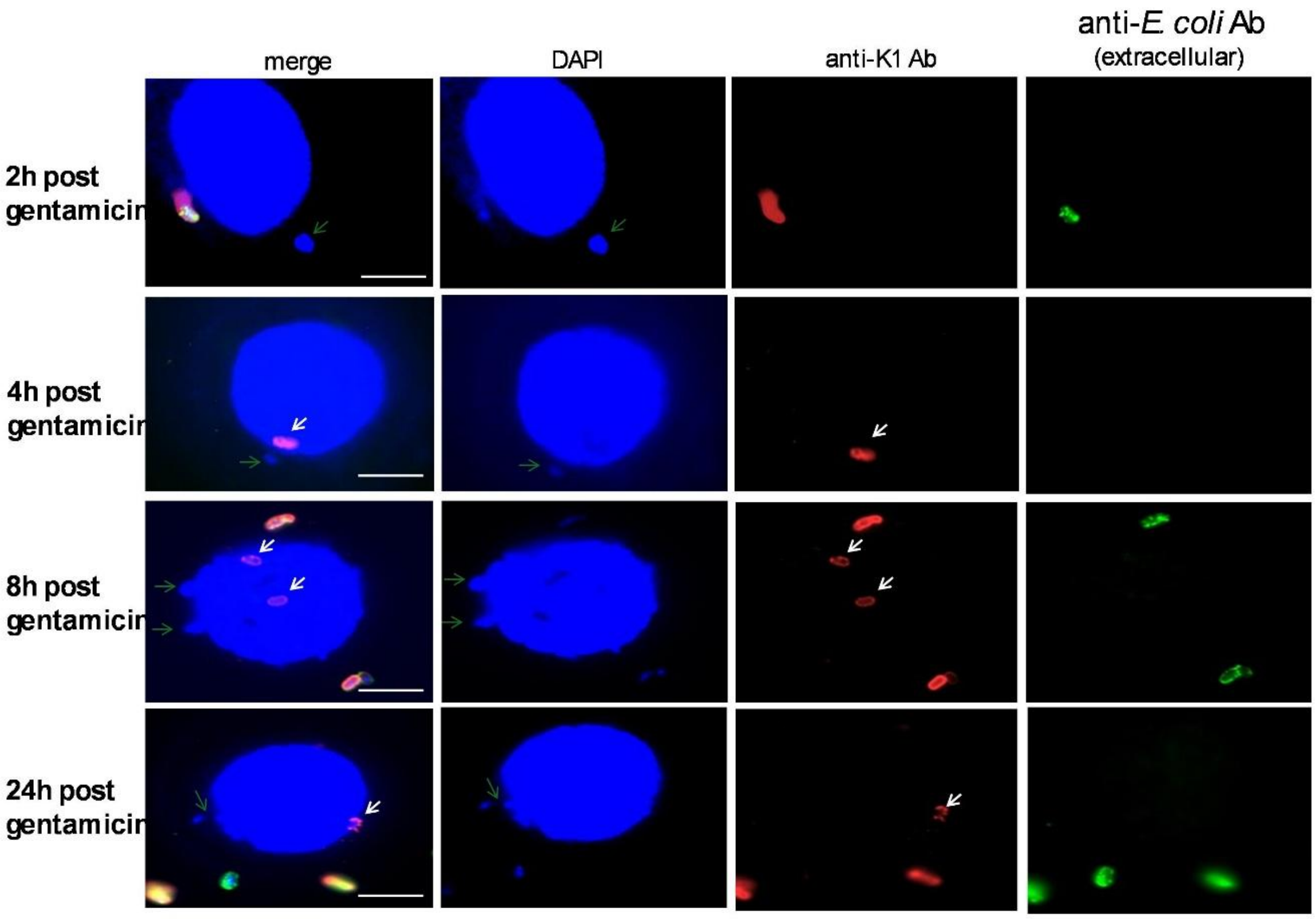

Figure S3. Capsule expression dynamics of FW-89 inside PD07i bladder cells.
PD07i cells were infected with FW-89 for 2 h, followed by gentamicin treatment to eliminate extracellular bacteria. Cells were incubated for the indicated times before staining. Extracellular bacteria were labeled with anti-E. coli antibody prior to fixation and permeabilization. Intracellular bacteria were subsequently stained with anti-K1 antibody, DAPI, and appropriate secondary antibodies (see Materials and Methods). Fluorescence microscopy images show individual channels and the merged composite. Scale bar = 10 µm. White arrows indicate encapsulated intracellular bacteria, and green arrows indicate intracellular unencapsulated bacteria. The experiment was performed three times, with the entire slide screened for intracellular events at each time point.

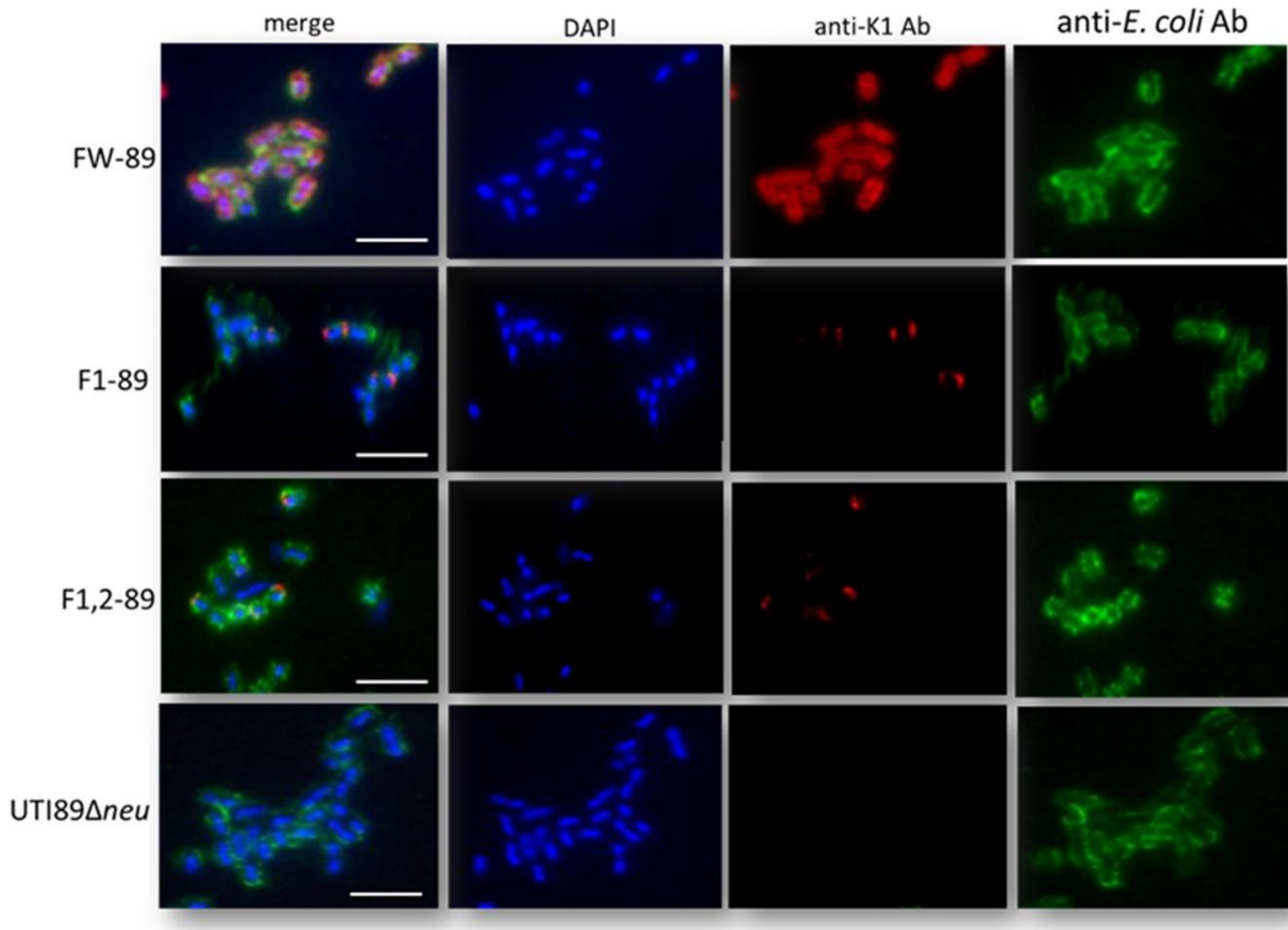

Figure S4. PR1-1 and PR1-1,2 mutants do not become fully encapsulated under infection-like conditions. Bacteria (FW-89, F1-89, F1,2-89, UTI89Δ*neu*) were incubated in Epilife media at 37°C, 5% CO_2 for 6 h, then stained with anti-E. coli, anti-K1, DAPI, and secondary antibodies as in PD07i infections. Fluorescence images show individual channels and composite. Scale bar = 10 µm.

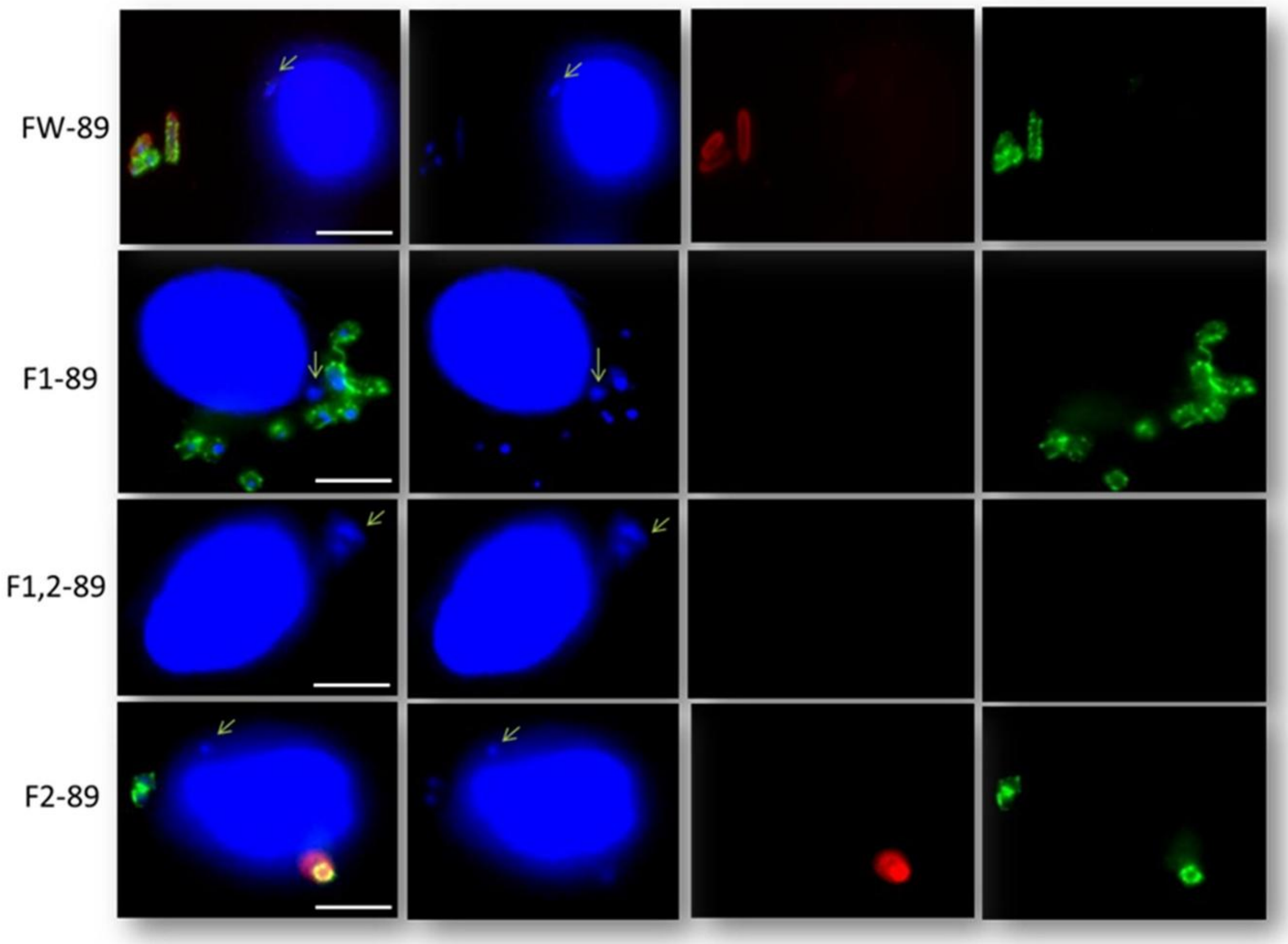

Figure S5 Initial PD07i cell invaders are unencapsulated bacteria (FW-89, F1-89, F1,2-89, F2-89). Cells were infected for 2 h, treated with gentamicin for 2 h, then labeled. Extracellular bacteria were stained before fixation; intracellular bacteria were stained with anti-K1, DAPI, and secondary antibodies. Fluorescence images show unencapsulated intracellular bacteria (green arrows). Scale bar = 10 µm. The experiment was performed two times and the whole slide was screened for any intracellular event at each time.

PR1-1 (-35) PR1-1 (-10)

-100 AATTGTTTTTGGTCTTAATAGTCAAGTGAGAAACATTTTAATAAATGATAA

-49 AAATCCTAAATTCCTTGTTCATAATGTAGGGGTGTGTTAAATAGGTGGG

+1 CAAAATTTGGTTCCCCTTCTCGCCTGTAATATTGCAGCATCTTCGCACCTCC

+53 ATGAGACATTGCGACTTAATAAGAAGGTGATAAGTCCTGCATATAAGCAT

PR1-2 (-35) PR1-2 (-10) PR1-3 (-35)

+103 GGACTGACCATGGTTTTATATTCATTAAAAATTTTGTTACAACCCATTGATT

PR1-3 (-10)

+155 TAGCATAAATAAATTATAGTGGGTTCGGGTTTGTTGTGACTGTGGCATTA

+205 TTTCCGTGCAAAGGAGCTGATATG

Figure S6. Predicted FNR-binding site relative to the region 1 (PR1) promoters. The predicted FNR consensus binding site within the PR1 regulatory region, which overlaps the predicted −10 element of PR1-2, is indicated in red. The transcription start site of each promoter (PR1-1, PR1-2, and PR1-3) is indicated by a bent arrow, and the corresponding +1 nucleotide is highlighted in bold green. The predicted −35 and −10 promoter elements are underlined. The nucleotides targeted for mutagenesis within the predicted −10 element of PR1-2 are highlighted in green. The kpsF start codon (ATG) is highlighted in yellow.

Table S1. Enumeration of intracellular encapsulated and unencapsulated bacteria within bladder epithelial cells at 24 h post-gentamicin treatment.

Strain	Number of bladder epithelial cells analyzed	Encapsulated intracellular bacteria (n)	Encapsulated bacteria per 100 bladder cells	Unencapsulated intracellular bacteria (n)	Unencapsulated bacteria per 100 bladder cells	Intracellular bacteria with polar polysaccharide staining (n)	Polar polysaccharide staining bacteria per 100 bladder cells
**FW-89**	112	6	5.4	0	0	0	0
**F1-89**	103	11	10.7	0	0	1	1.0
**F2-89**	120	24	20.0	0	0	0	0
**F1,2-89**	100	8	8.0	3	3.0	1	1.0
**F3-89**	21	1	4.8	6	28.6	0	0
**F2,3-89**	19	0	0	12	63.2	0	0

Note: Quantification was performed from one representative infection experiment. The intracellular infection assay was independently repeated at least three times, and representative images from these experiments are shown in Figures 7 and 8. Intracellular bacterial counts were normalized to 100 bladder epithelial cells to account for variation in the number of bladder cells analyzed. Unencapsulated intracellular bacteria were counted only when visualized within the perinuclear region or cytoplasm of bladder epithelial cells. Intracellular unencapsulated bacteria located within the nucleus could not be reliably distinguished from the DAPI-stained host nuclear material and were therefore not included in the counts.

Table S2. Enumeration of intracellular encapsulated and unencapsulated bacteria within bladder epithelial cells at 4 h post-gentamicin treatment

Strain	Number of bladder epithelial cells analyzed	Encapsulated intracellular bacteria (n)	Encapsulated bacteria per 100 bladder cells	Unencapsulated intracellular bacteria (n)	Unencapsulated bacteria per 100 bladder cells	Intracellular bacteria with polar polysaccharide staining (n)	Polar polysaccharide staining bacteria per 100 bladder cells
**FW-89**	91	15	16.5	8	8.8	0	0
**F1-89**	111	20	18.0	5	4.5	1	0.9
**F2-89**	100	12	12.0	2	2.0	0	0
**F1,2-89**	100	11	11.0	17	17.0	3	3.0
**F3-89**	91	3	3.3	25	27.5	0	0
**F2,3-89**	104	0	0	7	6.7	0	0

Note: Quantification was performed from one representative infection experiment. The intracellular infection assay was independently repeated at least three times, and representative images from these experiments are shown in Figures 7 and 8. Intracellular bacterial counts were normalized to 100 bladder epithelial cells to account for variation in the number of bladder cells analyzed. Unencapsulated intracellular bacteria were counted only when visualized within the perinuclear region or cytoplasm of bladder epithelial cells. Intracellular unencapsulated bacteria located within the nucleus could not be reliably distinguished from the DAPI-stained host nuclear material and were therefore not included in the counts.
